# Olfactory impairment in frontotemporal dementia: A systematic review and meta-analysis

**DOI:** 10.1590/1980-57642018dn13-020003

**Published:** 2019

**Authors:** Maren de Moraes e Silva, Camila Poletto Viveiros, Nikolai José Eustátios Kotsifas, Alexia Duarte, Evelyn Dib, Pilar Bueno Siqueira Mercer, Renata Ramina Pessoa, Maria Carolina Zavagna Witt

**Affiliations:** 1 Neurology Department, Hospital da Cruz Vermelha Brasileira Filial Paraná, Curitiba, PR, Brazil.; 2 Faculdade Evangélica do Paraná, Curitiba, PR, Brazil.; 3 Universidade Federal do Paraná, Curitiba, PR, Brazil.; 4 Faculdades Pequeno Príncipe, Curitiba, PR, Brazil.

**Keywords:** frontotemporal dementia, olfaction disorders, cognition, frontotemporal lobar degeneration., demência frontotemporal, transtornos do olfato, cognição, degeneração lobar frontotemporal.

## Abstract

Frontotemporal dementia (FTD) presents clinically in three variants: one behavioral and two with progressive primary aphasia - non-fluent/agrammatic and semantic. Defined by the degenerative process and cerebral atrophy, olfactory dysfunction occurs in up to 96% of previous FTD case series.

**Objective:**

the present study aims to critically synthesize data about the relationship between FTD and olfactory impairment to analyze the usefulness of olfactory evaluation tests as a complementary element in early diagnosis.

**Methods:**

a database search was performed using the keywords “olfactory OR smell OR olfaction AND frontotemporal dementia”. We included studies that evaluated olfactory function in patients diagnosed with frontotemporal dementia, all subtypes, compared with age-matched healthy controls. For comparative purposes, the effect size was calculated using Cohen’s D. The studies selected were categorized according to dementia variant and olfactory test type. A meta-analysis was performed using forest plots - homogeneity was evaluated by statistical tests (i^2^ and Cochran Q).

**Results:**

ten articles met the inclusion criteria. Heterogeneity was classified as low for semantic dementia olfactory identification and behavioral variant olfactory discrimination groups (i^2^ = 0 and 3.4%, respectively) and as moderate for the behavioral variant olfactory identification group (i^2^ = 32.6%).

**Conclusion:**

patients with the frontotemporal dementia behavioral variant seem to present with alterations in odor identification, but with preserved discrimination. Scent identification also seems to be impaired in semantic dementia. Therefore, we conclude that olfactory evaluation in these patients is possibly impacted by cognitive alterations and not by sensory deficits. Application of olfactory tests may prove important in differentiating prodromal states from other types of dementia with more pronounced olfactory impairment.

## RESUMO.

A demência frontotemporal apresenta-se clinicamente em três variantes: uma comportamental e duas com afasia progressiva primária - não fluente/agramática e semântica. Definida pelo processo degenerativo e atrofia cerebral, apresenta uma prevalência de disfunção olfatória de até 96% em séries anteriores.

**Objetivo:**

o presente estudo objetiva sintetizar criticamente dados sobre a relação entre DFT e o comprometimento olfatório para analisar a utilidade dos testes de avaliação olfatória como elemento complementar no diagnóstico precoce.

**Métodos:**

uma pesquisa de banco de dados foi realizada usando as palavras-chave *“olfactory OR smell OR olfaction AND frontotemporal dementia”.* Foram incluídos estudos que avaliaram a função olfatória em pacientes com diagnóstico de demência frontotemporal, todos os subtipos, em comparação com controles saudáveis ​​pareados por idade. Para fins de comparação, o tamanho do efeito foi calculado usando D de Cohen. Os estudos selecionados foram separados por variante de demência e tipo de teste olfativo. Uma meta-análise foi realizada utilizando gráficos floresta - sua homogeneidade foi avaliada por testes estatísticos (i^2^ e Cochran Q).

**Resultados:**

dez artigos preencheram os critérios de inclusão. A heterogeneidade foi classificada como baixa para os grupos de identificação olfatória em demência semântica e discriminação olfatória em variante comportamental (i^2^ = 0 e 3.4%, respectivamente) e moderada para identificação olfatória no grupo de variante comportamental (i^2^ = 32.6%).

**Conclusão:**

pacientes com variante comportamental de demência frontotemporal parecem apresentar alterações na identificação de odores, com discriminação preservada. A identificação de odores parece estar prejudicada, também, na demência semântica. Desta forma, concluímos que a avaliação olfatória nesses pacientes é possivelmente impactada por alterações cognitivas e não por déficits sensoriais propriamente. A aplicação de testes olfatórios pode ser importante na diferenciação de estados prodrômicos de outros tipos de demência com comprometimento olfatório mais pronunciado.

The physiological aging process often leads to decrease in olfactory function. About half of the population aged between 65 and 80 years have dysfunction in this system.^1^ Olfactory stimulus recognition is dependent on multiple processes, with odor detection being a function of peripheral structures and the olfactory bulb, while odor discrimination and identification are provided by cortical structures located in frontal and temporal lobes.^2^ These changes are present not only in elderly individuals, but also across the spectrum of neurodegenerative diseases, such as Alzheimer’s and Parkinson’s disease.^3^^,^ 4 Olfactory dysfunction in patients with dementia affects not only the performance of daily activities, but also the palate, which may influence appetite and lead to dietary restrictions.^5^

Frontotemporal dementia (FTD) is a neurodegenerative pathology with involvement of frontal, temporal, or both lobes.^6^ This dementia mainly affects individuals aged between 45 and 60 years and is associated with behavioral changes, although other cognitive functions, such as memory and visuospatial abilities, are affected to a lesser degree.^6^ FTD presents clinically in three variants: one behavioral and two with progressive primary aphasia - non-fluent/agrammatic and semantic. Defined by a degenerative process and cerebral atrophy, olfactory dysfunction occurs in up to 96% of FTD cases.^7^ Olfactory system neuroanatomy - with parahippocampal gyrus and entorhinal area involvement - and the cortical impairment explain the prevalent impairment of olfactory function in this dementia.^5^

Although there are no specific olfactory involvement patterns for diagnosing the different types of dementia, assessment of olfactory alterations proves to be a valuable tool in aiding diagnosis and serving as a biomarker of disease progression.^8^ In this context, the present study aims to critically synthesize data found in the literature evaluating the relationship between FTD and olfactory impairment by applying olfactory evaluation tests to analyze their usefulness as a complementary element in early diagnosis.

## METHODS

The present study protocol is registered in the PROSPERO database under registration code CRD42018095155.

### Literature search

A search of the MEDLINE, SciElo, PubMed and LILACS databases using the keywords “olfactory OR smell OR olfaction AND frontotemporal dementia” was performed by four researchers - individually and blinded. The search was completed on June 19, 2018.

We included studies that evaluated olfactory function in patients diagnosed with frontotemporal dementia, all subtypes, compared against age-matched healthy controls. Cross-sectional and longitudinal articles were included, published in Portuguese, English or Spanish, without publication date restriction. Editorials, reviews, guidelines and letters were excluded. Articles that did not clearly state which diagnostic criteria were used or that had no control group were also excluded. Numerical data necessary for meta-analysis that were unclear in the article and that could not be calculated were requested from the corresponding author by email articles by authors who did not provide the requested data within 30 days were excluded.

### Evaluation and selection

An initial search was conducted based on titles and abstracts - for articles whose abstracts were not sufficiently informative, the full texts were assessed at this stage. We separated papers that initially met inclusion and exclusion criteria. In a second step, full texts were read, with subsequent selection of those that, besides meeting the criteria, provided enough data for analysis. Missing data were requested from corresponding authors. Disagreements among the researchers were resolved by consensus after evaluation by an additional researcher, where agreement level was calculated by a *kappa* value.

### Data analysis

For purposes of comparison, the effect size was calculated using Cohen’s D.^9^ The effect was considered low for results <0.2, intermediate for 0.2-0.5, and high for ≥0.8. For summarization, the random effects method was used.

Studies included in the meta-analysis were summarized using forest plots and their homogeneity assessed using Cochran’s Q statistics^10^ and I^2^. Statistical analysis was performed using R® software. Comparison between continuous variables was done using the Mann-Whitney test.

### Publication bias evaluation

Evaluation of publication bias was carried out by a combination of methods: funnel plot visual analysis, Rosenthal’s “Fail safe N”^11^ method, and Duval and Tweedie’s “Trim and fill”^12^ method.

### Categorization of studies

Selected studies were initially categorized by dementia subtype. For the purposes of meta-analytic evaluation, two groups were considered: semantic dementia and behavioral variant. Agrammatic Primary Progressive Aphasia was not included due to lack of studies.

In addition, a further subdivision was performed according to olfactory analysis: identification and discrimination tests. “Identification” has been defined by studies as the ability to identify the source of an olfactory stimulus and naming it, while “discrimination” refers to olfactory thresholds.

The information in articles that addressed more than one variant, and/or more than one type of olfactory analysis, was also subdivided for separate analysis. Studies that involved mixed groups without dementia subtype discrimination were included in the Discussion, but excluded from meta-analytic summary.

## RESULTS

### Studies selected

**Figure 1:**
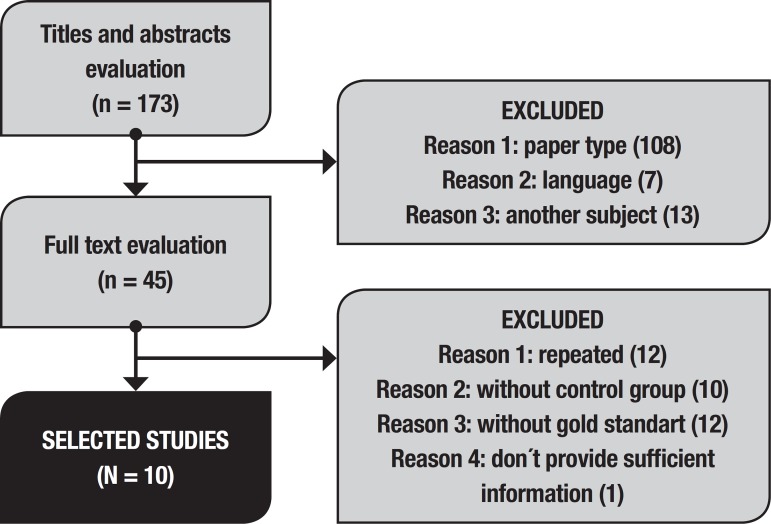
Study selection flowchart.

Eleven articles fulfilled criteria for inclusion in this study, but of these, one had missing information precluding its inclusion in this analysis. Article selection is depicted in the flowchart (Figure 1). Concordance had a kappa of 0.72.

### Study characteristics and effect size

The studies selected were published between 2006 and 2017, and carried out in Europe or the United States of America. No studies addressing the subject in question were conducted elsewhere.

The studies selected and their characteristics are shown in Table 1, together with the effect size calculated for each sample.

**Table 1:** Characteristics of studies.

Study	Variant	Country	Diagnostic criteria	FTD (n)	Control (n)	Tool	Modality	FTD Score	Control Score	Cohen’s D
Luzzi et al. 2006 (a1)	SD	Italy/UK	Neary et al. (1998)	8	20	OPSB	Identification	6	15	4.02
Luzzi et al. 2006 (a2)	BV	Italy/UK	Neary et al. (1998)	11	20	OPSB	Identification	9	10	3.5
Luzzi et al. 2006 (b1)	SD	Italy/UK	Neary et al. (1998)	8	20	OPSB	Discrimination	14	14	0
Luzzi et al. 2006 (b2)	BV	Italy/UK	Neary et al. (1998)	11	20	OPSB	Discrimination	14	14	0
McLaughlin et al. 2007	Mixed	USA	Neary et al. (1998)	14	14	BSIT	Identification	7.0	10.4	1.53
Rami et al. 2007 (a)	Mixed	UK	McKhann et al.	3	4	UPSIT	Identification	15.3	29.5	4.4
Rami et al. 2007 (b)	Mixed	UK	McKhann et al.	3	4	UPSIT	Discrimination	12.33	16	2.58
Piwnica-Worms et al. 2010	SD	UK	Neary et al. (1998)	4	6	UPSIT	Identification	20.25	31.83	2.61
Omar et al. 2013 (1)	BV	UK	Neary et al. (1998)	12	17	UPSIT	Identification	16.6	34.7	2.87
Omar et al. 2013 (2)	SD	UK	Neary et al. (1998)	8	17	UPSIT	Identification	17.5	34.7	3.35
Magerova et al. 2014 (1)	SD	Czech Republic	Neary et al. (1998)	6	15	MHST	Identification	10.25	14.8	1.49
Magerova et al. 2014 (2)	BV	Czech Republic	Neary et al. (1998)	9	15	MHST	Identification	10.71	14.8	1.65
Orasji et al. 2016 (a)	BV	Holland	Neary et al. (1998)	9	11	BSIT	Identification	6.7	8	0.48
Orasji et al. 2016 (b)	BV	Holland	Neary et al. (1998)	9	11	ODT	Discrimination	11.1	11.8	0.23
Markopoulou et al. 2016	Park	USA/ Poland/ Germany	Genetic	21	3	UPSIT	Identification	13.32	32.33	2.58
Pilotto et al. 2016 (a)	BV/ALS	Germany	Strong et al.	17	11	Sniffin’ Sticks	Identification	9.9	11.9	1.85
Pilotto et al. 2016 (b)	BV/ALS	Germany	Strong et al.	17	11	Sniffin’ Sticks	Discrimination	11.4	13.9	1.67
Perry et al. 2017	BV	USA	Rascovsky et al. (2011)	19	21	Homegrown	Discrimination	0.69	0.87	1.12

SD: semantic dementia; BV: behavioral variant; Park: with Parkinsonism; BV/ALS: behavioral associated with amyotrophic lateral sclerosis; UK: United Kingdom; USA: United States of America; OPSB: odor perception and semantics battery; BSIT: brief smell identification test; UPSIT: University of Pennsylvania Smell Identification Test; MHST: Motol Hospital Smell Test; ODT: Odor Discrimination Task.

The effect size obtained for each study was high, with the exception of the study by Orasji et al.,^13^ which showed a moderate effect.

### Olfactory tests

A total of seven different olfactory assessment tools were applied by the selected studies. The most frequently used was the UPSIT (University of Pennsylvania Smell Identification Test), employed by 4 studies. The other tools were used only by 1 study each. The UPSIT consists of a multiple-choice test for 40 different odorants. The BSIT (Brief Smell Identification Test) is a shortened form of this test, containing only 12 items. The Sniffin Sticks tool, also frequently used in the literature, evaluates olfaction by means of 12 odorants, having 4 possible answers placed on cards. The Odor Perception and Semantics Battery (OPSB) comprises five tasks with 16 odors: odor discrimination, picture matching, naming and two control tests. The Motol Hospital Smell Test (MHST) is a multiple-choice identification test with 18 essential oils developed at the Motol Hospital Memory Clinic, tested in Czech patients, with a 4-choice list response.

Adaptations of the images was carried out to allow adequate evaluation of semantic dementia patients.

### Characteristics of samples

By summarizing the sample diagnosed with the behavioral variant, we obtained a sample of 77 patients, 26 patients with semantic dementia and 175 healthy controls. Two studies used a mixed sample, not reporting the variant, involving a total of 17 patients. Sample characteristics in terms of age distribution, gender, education and Mini-Mental State Examination performance are given in Table 2.

**Table 2:** Characteristics of samples.

Study	Variant	FTD age	Control age	MMSE	FTD Male %	Control Male %	Education
Luzzi et al. 2006 (a1)	SD	68	65	21	62.5	62.5	14
Luzzi et al. 2006 (a2)	BV	64	65	24	72.7	50	10
Luzzi et al. 2006 (b1)	SD	68	65	21	62.5	62.5	14
Luzzi et al. 2006 (b2)	BV	64	65	24	72.7	50	10
McLaughlin et al. 2007	Mixed	64.9	65.9	20.7	42.9	42.9	12.8
Rami et al. 2007 (a)	Mixed	70	69.5	23.6	100	100	NI
Rami et al. 2007 (b)	Mixed	70	69.5	23.6	100	100	NI
Piwnica-Worms et al. 2010	SD	59	61.5	NI	66.6	66.6	NI
Omar et al. 2013 (1)	BV	66.1	66.2	23.5	100	47.1	NI
Omar et al. 2013 (2)	SD	66.1	66.2	22.8	87.5	87.5	NI
Magerova et al. 2014 (1)	SD	66.3	66.9	21.8	66.7	26.7	NI
Magerova et al. 2014 (2)	BV	63.11	66.9	24.11	26.7	33.3	NI
Orasji et al. 2016 (a)	BV	73.1	71.6	25	88.8	54.5	2.1
Orasji et al. 2016 (b)	BV	73.1	71.6	25	88.8	54.5	2.1
Markopoulou et al. 2016	Park	46.5	51.6	NI	46	46	NI
Pilotto et al. 2016 (a)	BV/ALS	71.4	64.3	25.7	45.4	25.7	5.9
Pilotto et al. 2016 (b)	BV/ALS	71.4	64.3	25.7	45.4	25.7	5.9
Perry et al. 2017	BV	63.9	61.7	24.4	64	31	NI

SD: semantic dementia; BV: behavioral variant; Park: with Parkinsonism; BV/ALS: behavioral associated with amyotrophic lateral sclerosis; MMSE: Mini-Mental State Examination; NI: not informed.

Of the analyzed studies, four did not report presence of smokers in the sample^14^^-^ 17 while four reported this characteristic as an exclusion criterion.^18^^-^ 21 The other study samples comprised up to 40% smokers.^18^^,^ 22

### Characteristics of semantic dementia samples

Regarding studies that addressed semantic dementia, only one evaluated olfactory discrimination, in a sample of 28 individuals.^18^ Studies that evaluated this variant by olfactory identification involved a total sample of 84 individuals, from four studies included.

In relation to studies that evaluated odor identification, mean age was 64.86 ± 3.99 years for the dementia group and 64.48 ± 2.41 years for the control group. The difference between these samples was not statistically significant (p = .08857). Analysis of gender distribution revealed 70.82% ± 3.99 males in the dementia group and 60.82% ± 25.26 in the control group, with no statistically significant difference between samples (p = .6286).

The mean score on Mini-Mental State Examination was 21.87 ± 0.90. Only one study reported the average educational level of the sample (14 years).^18^ Statistical correlation between performance on cognitive screening and olfactory evaluation was not possible due to the small number of studies included in analysis.

### Characteristics of behavioral variant samples

Summarizing of the behavioral variant sample resulted in 132 individuals for odor identification and 125 for odor discrimination tests. Results of the comparison between sample demographics, Mini-Mental State Exam ination performance and education are shown in Table 3.

**Table 3:** Comparison between behavioral variant samples.

	Identification		p	Discrimination		p
	FTD (n 58)	Control (n 74)		FTD (n 56)	Control (n 63)	
Age (mean, years)	67.54 ± 4.47	66.81 ± 2.87	.8413	68.10 ± 4.84	65.65 ± 3.95	.8857
Male (% mean)	66.72 ± 30.37	42.12 ± 12.11	.3095	67.73 ± 18.09	40.30 ± 14.09	.1142
MMSE (mean)	24.46 ± 0.88	–	–	24.77 ± 0.74	–	–
Education (mean, years)	6.00 ± 3.95		–	6.00 ± 3.95		–

MMSE: Mini-Mental State Examination.

### Summary

A summary was performed for the following categories: identification tests in the behavioral variant, identification tests in the semantic variant and discrimination tests in the behavioral variant. The number of studies in the other subcategories was insufficient for adequate analysis.

**Figure 2:**
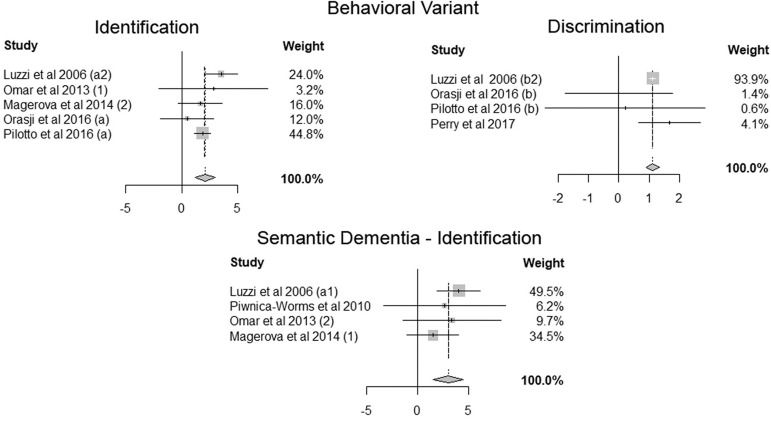
Summary of studies.

Heterogeneity was classified as low for semantic dementia olfactory identification and behavioral variant olfactory discrimination groups (i^2^ = 0 and 3.4%, respectively) and moderate for the behavioral variant olfactory identification group (i^2^ = 32.6%). A forest plot summarizing the studies in the three categories is depicted in Image 2.

The difference between identification and discrimination tests for the behavioral variant was not statistically significant (p = .0556).

### Publication bias evaluation

A funnel plot revealed an asymmetry on visual analysis, which was analyzed by linear regression (p = .0468). The “Fail-Safe N” method revealed the need for 1148 studies to be incorporated to make findings insignificant (p <.0001). However, using the “Trim and fill” method, it was found that 6 studies were needed to ensure symmetry of the funnel plot (p <.0001).

## DISCUSSION

Olfactory function impairment is a widely studied feature in several neurodegenerative diseases,^2^ but remains little explored in the context of FTD. It is known that patients with this dementia present degeneration of important areas for smell processing, as well as changes in the reward system.^14^ There is a correlation between performance in odor identification and executive control in these patients.^18^ In addition, there is evidence that individuals with olfactory changes in FTD are insensitive to negative information, that is, unpleasant odors become less aversive. This is due to degeneration of components related to emotional interpretation of sensation (such as ventral insula and amygdala).^14^

It is important to bear in mind that FTD patients may have difficulties identifying odors secondary to semantic impairment, which may require adaptation in classically used tests so that they serve the purpose of purely sensory evaluation. Use of images may serve to provide more accurate application of these tests in this population.^17^

After the adaptations had been made, based on our review, a positive correlation was evident between FTD and odor identification in both the behavioral and semantic variants. The role of temporal olfactory cortical areas in processing this type of stimulus can serve as a factor for justification when analyzing these results, taking into account the pattern of atrophy presented in this disease.^20^ Also, studies that evaluated odor discrimination in the behavioral variant failed to find alterations in the dementia group, demonstrating that there was no impairment in this olfactory modality. Only one paper evaluated odor discrimination in semantic dementia, with results in agreement with the other studies, finding no difference between the case and control groups.^18^ The failure to find a statistically significant difference between the two test modalities is probably due to the low number of studies included, since the p-value was borderline.

Studies that indicated absence of olfaction impairment suggest there may be an impairment of association between the odor presented and the semantic knowledge, whereby alterations seen on tests may not be olfactory essentially, but due to association impairment.^13^ However, this hypothesis is not supported by studies indicating that olfactory impairment is of a similar magnitude in different subtypes of frontotemporal dementia.^19^ This finding may suggest that the pathophysiology is not related to impaired semantic ability alone, else greater olfactory loss would be expected in semantic dementia. Another point to be taken into consideration regarding the studies evaluated is the small sample size, which may impact the statistical significance of findings.

In relation to odor identification in the semantic variant, patients with this diagnosis had significantly worse mean results than the control groups. This corroborates the hypothesis that “multimodal” semantic degeneration occurs (not restricted to linguistics), helping to elucidate the mechanisms by which it occurs, although further studies are still needed in this area.^17^ The findings discussed here also converge with the theory of influence of temporal limbic area involvement in patients with semantic dementia.^19^

It is noteworthy that only five studies had smoking as an exclusion criterion. Smoking promotes a greater risk for olfactory dysfunction.^23^ Thus, this factor should be considered in sample selection for further studies.

Given the consistency of results, an additional tool could be developed to evaluate dementia patients based on olfactory changes. However, these impairments also manifest in Alzheimer’s disease,^3^ Amyotrophic Lateral Sclerosis^21^ and constitute one of the most well-described early signs of Parkinson’s disease in the literature.^4^ Thus, although more studies are needed to accurately determine olfactory impairment characteristics in each condition, there is clearly a relationship between these alterations and various neurological diseases. Adequate characterization of olfactory involvement patterns in dementias can aid differential diagnosis and early detection of non-cognitive symptoms.

This study is limited by the low number of papers in the literature addressing the subject, especially in relation to semantic dementia. In addition, the large variability in olfactory tests used by the studies may in some way impact interpretation of the results, where further studies are needed to compare different olfactory evaluation tools.

In conclusion, patients with frontotemporal dementia - behavioral variant seem to exhibit alterations in odor identification, but with preserved discrimination. Scent identification also seems to be impaired in semantic dementia, but more studies are needed to confirm the pattern in this variant. Therefore, we conclude that olfactory evaluation in these patients is possibly impacted by cognitive alterations and not only by sensory deficits. Application of olfactory tests may prove important in differentiating prodromal states from other types of dementia with more pronounced olfactory impairment.
